# Screening for mild cognitive impairment in people with obesity: a systematic review

**DOI:** 10.1186/s12902-021-00898-0

**Published:** 2021-11-17

**Authors:** Nimantha Karunathilaka, Sarath Rathnayake

**Affiliations:** 1 Department of Nursing and Midwifery, Faculty of Allied Health Sciences, General Sir John Kotelawala Defence University, Ratmalana, Sri Lanka; 2 Department of Nursing, Faculty of Allied Health Sciences, University of Peradeniya, Peradeniya, Sri Lanka

**Keywords:** General population, Mild cognitive impairment, Obesity, Screening

## Abstract

**Objective:**

Recent evidence demonstrates that obesity is associated with developing cognitive impairment. However, evidence related to the assessment of mild cognitive impairment (MCI) in people with obesity is limited. Therefore, this systematic review aimed to examine evidence concerning the screening of MCI in people with obesity from the general population.

**Method:**

We conducted a systematic search of CINHAL, EMBASE, MEDLINE, PsycINFO and PubMed electronic databases for observational studies to assess MCI in people with obesity from the general population. PRISMA guideline was followed. The articles published from January 2011 to July 2021 were included.

**Results:**

Database search found 3104 sources. After the screening process, two articles from China and Egypt were included. The main age groups assessed were middle-aged adulthood and older adulthood. There were no studies undertaken in young adults or across the life span. Obesity was assessed by body mass index. MCI was assessed by cognitive screening tools; Mini-mental State Examination and Addenbrooke’s Cognitive Examination. The prevalence of MCI in people with obesity was 18.5 % and 42.9 % in Chinese and Egyptian studies, respectively. Only one study supported a positive association between MCI and obesity.

**Conclusions:**

Limited studies were found on screening MCI in people with obesity in the general population. The available evidence was not adequate to explain the overall prevalence, possible associations, and the best tool for assessing MCI in people with obesity. Expanding screening studies for MCI in people with obesity in the general population is essential.

## Introduction

Obesity is a complex and multifactorial but preventable disease [1], and around one-third of the world population is considered overweight or obese [2]. Obesity is defined as abnormal and excessive fat accumulation that presents a risk to health [1, 2]. Regardless of age, gender, geography or socioeconomic status, there is an increasing trend of obesity [3, 4]. Obesity leads to many non-communicable diseases, for example, diabetes, cardiovascular diseases, and cancer [2, 5]. Additionally, evidence indicates that obesity is associated with the development of cognitive impairment and has a higher potential for developing dementia in the mid and later life of individuals [6–8]. Several pathophysiological mechanisms explain the influence of obesity in cognitive impairment. Obesity reduces neural integrity, including atrophy of grey and white matter, shrinking the hippocampus, and reducing prefrontal cortex volume, contributing to cognitive impairment [7, 9]. Furthermore, central and systemic inflammation, the over-activation of microglia and astrocytes, and blood-brain barrier dysfunction also play a significant role in cognitive impairment among people with obesity [9–12].

Age-related cognitive decline is a normal biological process in humans [13] and is independently associated after adjusting the major neuropathological factors in later life [14]. Additionally, mild cognitive impairment (MCI) is the stage of cognition between normal cognition and dementia and is the first sign of the alteration of cognition [15–17]. Usually, MCI can be reversed to normal cognition, while dementia is the permanent damage of neural activities [15, 16]. Therefore, early identification of MCI is essential to plan strategies to promote health and sminimise the risk of developing dementia.

Obesity is classified as sgeneralised and central obesity. sGeneralised obesity is assessed by body mass index (BMI), and central obesity is assessed by waist-to-hip ratio (WHR) [18]. Obesity, including sgeneralised or central obesity or both, has been positively associated with cognitive impairment in young [8] and middle adulthood [19–23]. However, Vidyanti et al. [24] and Pedditizi et al. [25] reported that older age is less frequently associated with cognitive impairment in people with obesity. Although a number of studies are available to assess cognitive impairment in people with obesity, there is little attention to the examination of MCI in people with obesity from the general population. Additionally, the majority of studies have been conducted among obese people with specific disease conditions such as diabetes [26], cardiovascular disease [27], obstructive sleep apnea [28] and HIV infection [29]. Furthermore, some studies do not support the identification of MCI as they have focused on the identification of overall impairment of cognitive functions [30–32]. Furthermore, there was a lack of evidence in selecting the best MCI screening tool for the disease-specific cohort as well as various study designs [26–29]. Therefore, the present study aimed to critically review the available literature on screening of MCI in people with obesity from the general population.

## Methods

A systematic review was conducted to gather the available evidence for screening of MCI among people with obesity in the general population. The main research question was formulated using the PICO/PIO method, where “P” stood for the study population i.e., people with obesity in the general population, including a high body mass index (sgeneralised obesity) or a high waist-to-hip ratio (central or abdominal obesity) and excessive adiposity. “I” stood for intervention, which was the screening of MCI, while outcome “O” was referred to as identifying MCI of people with obesity. Consequently, the following review questions were addressed in this systematic review:

1. What is the prevalence of MCI in people with obesity in the general population?
2. What are the cognitive assessment tools that can be used for screening mild cognitive impairment among people with obesity?

We searched five electronic databases: CINHAL, EMBASE, MEDLINE, PsycINFO and PubMed. The search terms were scategorised into two conceptual areas: “Body Mass Index” OR “Waist-to hip-ratio” OR “Waist-to-hip ratio” OR Adiposity [MeSH] OR Obesity [MeSH] AND “Mild cognitive impairment” OR “Mild cognitive dysfunction*” [MeSH] OR “Mild mental deterioration” OR “Mild cognitive decline” OR “Neurocognitive disorder*” [MeSH]. Keyword searches were performed on title, abstract and keywords using Boolean operators. The Preferred Reporting Items for Systematic Reviews and Meta-Analyses (PRISMA) guidelines were followed in the screening process [33]. Moreover, the reference lists of selected full-text articles were screened further to find any additional relevant articles, and none were found.

The inclusion criteria for the review were studies that included human subjects aged 18 and over and peer-reviewed journal articles published in English from January 2011 to June 2021. Moreover, MCI should be screened in the people with obesity from the general population or community. Additionally, observational studies were included for this review, including cohort studies, case-control studies, and cross-sectional studies. We excluded studies that focused on screening obesity in people who were already diagnosed with MCI, cognitive impairment, and any other psychological disorders. Articles that did not state local or internationally accepted cut off values to define obesity (sgeneralised or central), and the subjects who have been diagnosed with metabolic syndrome were also excluded. Moreover, experimental studies, case reports, case series, review articles, editorials, and commentaries were excluded.

**Fig. 1:**
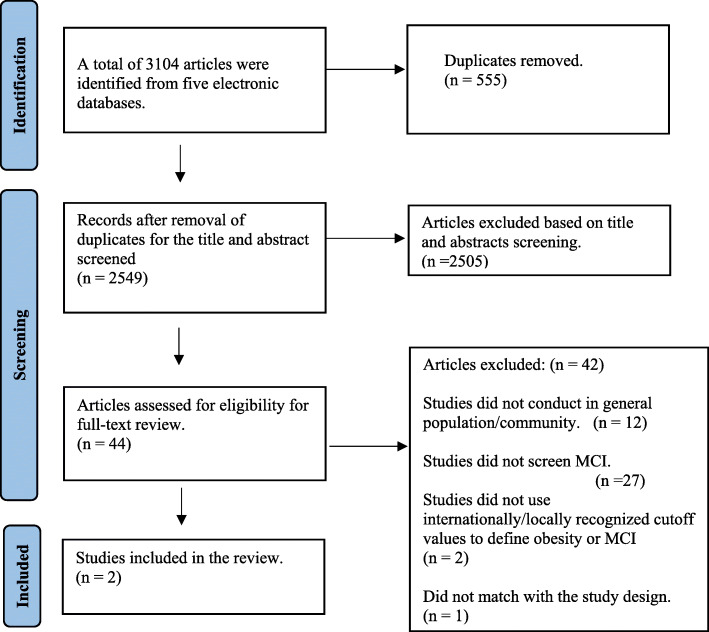
Flow chart of study selection; Adapted from Page et al., [33]

The literature search was combined with mesh terms, specific terms, and keywords. There were 3104 articles, including 502 from PubMed, 1876 from EMBASE, 483 from Medline, 112 from CINHAL and, 131 from PsycINFO. After removing 555 duplicates, 2549 articles were selected for titles and abstracts screening. Both authors (NK & SR) screened 10 % of sources (255 articles) for titles and abstracts based on the given inclusion and exclusion criteria, and the consensus was achieved. Then, NK continued the title and abstract screening. Based on the title and abstract review, 2505 articles that did not meet the study inclusion criteria were excluded. A total of 44 articles were included in the full-text review. Initially, two authors reviewed the content of the full-text articles. The average initial kappa value was 0.645. Discrepancies were discussed, and a consensus was achieved. Finally, two articles that satisfied the inclusion criteria were included for the final analysis (Fig. 1).

Information about all included studies was tabulated under the following: author, year, location, the aim of the study, study design, participant age, sample size, obesity assessment method, data collection tools, and main outcomes. Furthermore, extracted information was rearranged based on the type of obesity assessment and age categories to evaluate the better patterns of cognitive screening tools. The methodological quality of the studies was assessed by Jonna Briggs Institute (JBI) critical appraisal tools [34]. Meta-analysis was not possible because the selected two studies can not be meaningfully pooled, and their results were not sufficiently similar, increasing the heterogenicity of the pooled results [35]. Furthermore, the protocol of this systematic review was registered in the PROSPERO website (CRD42021260547).

## Results

### Study characteristics

One study was a cross-sectional survey conducted in China [36], while the other was a case-control study conducted in Egypt [37]. The age groups were above 45 years to below 55 years [37] and ages over 60 to 100 years [36]. The sample size in the Chinese study was 3242 [36]. In the Egyptian study, 161 obese people participated as cases while 61 healthy people participated as the control group [37] (Table 1).

**Table.1:** Articles included in Systematic Review

Authors, Year, country	Aim of the study	Study Design	Participants’ Age (in years)	Sample Size	Obesity Assessment	Cognitive Screening Tool	Major Findings
Yuan et al., [36] China	To investigate gender and age as moderators in the association between BMI and mild cognitive impairment (MCI) among rural older adults	Cross-sectional	Aged 60 and above (Range from 60 to 100 ) Age scategorised into Below 75 and above 75 .	*n* = 3242	BMI [sCategorised into underweight (low BMI), normal weight (normal BMI), overweight (elevated BMI) and obese (high BMI)]	MMSE (30 Items; Chinese Version)	**Below 75 years** (Both men and women) There was no significant difference in MCI among overweight and obese categories when compared to normal BMI. (*p*>0.05) **Above 75 years.** Older men Compared to nomal BMI category, Overweight category had higher risk of MCI (aOR = 2.32, 95 % CI: 1.17–4.61; *p*<0.05) Older women BMI (overweight and Obesity) vs. MCI – NS
Salama et al., [37] Egypt	To assess MCI prevalence and its relation with lifestyle risk factors among obese adults	Case-control	Mean age case (Obese) – 52.1± 5 Mean age control (Healthy) – 51.3± 6	Case (Obesity) *n*=161 Control (Healthy) *n*=69	BMI Obesity Gr. I (BMI <35) Obesity Gr. II (BMI 35-39.9) Obesity Gr. III (BMI>40)	ACE (Version III)	MCI was assessed between Obesity categories (I, II and III) and control (healthy adults) MCI between Grade I and Control (OR – 5.5, 95 % CI: 2.2-13.5; *p*<0.001) MCI between Grade II and Control (OR – 6.8, 95 % CI: 2.7-16.9; *p*<0.001) MCI between Grade III and Control (OR – 4.8, 95 % CI: 1.8-12.5; *p*<0.001)

### Screening tools for obesity and MCI

In both studies, sgeneralised obesity has been assessed through BMI [36, 37]. The Chinese study is used the Asia Pacific cut off value to scategorised BMI into underweight, normal weight, overweight, and obesity [36, 38]. Egyptian study [37] has recruited obese people, and obesity is scategorised into obesity grades I, II and III: 30<BMI>34.99 (Grade I), 35<BMI>39.99 (Grade II), and BMI>40 (Grade III), respectively [39] (Table 1).

MCI has been assessed through the accepted cut-off values of Mini-Mental State Examination (MMSE) [36] and Addenbrooke’s Cognitive Examination (ACE III) [37] (Table 1). MMSE consists of orientation, memory, attention, language, and visuospatial sub-domains [40], while ACE consists of attention, memory, fluency, and language sub-domains [37]. The Chinese study has a different cut-off value for screening MCI in MMSE based on participant’s education level (MMSE cut-off <17 for illiteracy; MMSE cut-off <20 for up to primary education and, MMSE cut-off <24 for higher than primary education) [41]. In the Egyptian study, the cut-off value for screening MCI has been calculated based on the mean score of ACE III in healthy adults. The mean score of ACE III is 83. The cut-off value for screening MCI has been set below the mean score [42] (Table 1).

### Prevalence of MCI and its relationship with obesity

Yuan et al. [36] reported that the prevalence of MCI was 18.5 %. Furthermore, only older men (age over 75 years) who had elevated BMI had a higher risk of MCI compared to normal BMI (p<0.05). There was no significant association between elevated/higher BMI and MCI (p>0.05) in older men (age range from 60 to 75 yrs.) and women (age over 60 yrs.) [36] (Table 1). Salama et al., [37] revealed that the prevalence of MCI was 42.9 % among people with obesity (cases). Furthermore, MCI was varied among Grade I, II, III, and the control group as 42.2 %, 47.2 %, 38.6 %, and 11.6 %, respectively [37]. MCI score was significantly different in Grade I, II, and III categories when compared to the control (*p*<0.001) [37] (Table 1).

## Discussion

This systematic review aimed to evaluate the observational studies that screened MCI in people with obesity in the general population. We found that limited studies were available in the literature on assessing MCI in people with obesity in the general population. Our screening process identified that most of the studies were conducted among patients diagnosed MCI [43–45], cognitive impairment and dementia cohorts [46–48] and other disease specific populations, for example, diabetes, cardiovascular disease, obstructive sleep apnea and HIV infection [26–29]. Since only two studies examined MCI in people with obesity in the general population, meta-analysis was not meaningful as it was pooled in a single cross-sectional and case-control study, which increases the heterogenicity of the pooled results [35]. Furthermore, Mueller et al. [49] stated that there was scanty methodological guidance to perform systematic reviews and meta-analyses of observational studies. Therefore, it is difficult to make a precise conclusion on the prevalence of MCI, the relationship between MCI and obesity, and the best screening tools to screen MCI in obese people. Consequently, expanding studies to examine MCI in people with obesity in the general population is essential.

In the present review, the prevalence of MCI among people with obesity in the general population was 18.5 % (cross-sectional) and 42.9 % (case-control) in Chinese and Egyptian studies. However, Moretti et al., [50] and Lara et al., [51] stated that the prevalence of MCI in the general population in Italy and Span was around 6.0 % and 9.6. Therefore, it is suggested that the prevalence of MCI is higher among people with obesity [36, 37] than the prevalence of MCI in the general population [50, 51]. We further found that a study in China reported the prevalence of MCI among people with obesity in the general population as 21.8 % [52]. Additionally, stroke [50], depressive symptoms [50] or depression [51, 53], sleep disturbances [51], history of head injury [53], and lower educational status [53] are also potential associated factors for MCI in the general population. Consequently, early screening of MCI among people with obesity in the general population would be beneficial to mitigate further deterioration of cognitive function that leads to dementia in later life.

In line with the recent studies [24, 25], this study revealed that cognitive impairment was less frequently associated with older age. The present review supported that there was no relationship between MCI and older women (age over 60 years) and men (age below 75) with obesity. However, in middle adulthood, cognitive impairment among people with obesity was inconsistent. While the majority of studies revealed that there was a significant relationship between obesity and cognitive impairment [20, 21, 28, 51, 52, 54], only a few studies state that there was no such relationship [19, 22, 23]. However, the present review supports a significant relationship between MCI and obesity (Grade I, II, and III) during middle adulthood (p<0.05) [37]. Although there was no study among people with obesity within the age group of young adulthood, two studies conducted in the USA [8] and Iran [50] stated that there was a significant association between obesity and cognitive impairment. However, all these studies were conducted in the pre-defined?? population or disease-specific population and also did not state the cut-off value for mild, moderate, and severe cognitive impairment [53, 55–57].

Obesity is mainly assessed through sgeneralised and central obesity. Generalised obesity can be assessed through BMI and central obesity can be assessed by WHR [20, 21, 23]. In addition, fat mass, body fat percentage, and lipid accumulation products (LAP) are also used in estimating the level of obesity [52, 58]. Although in the present review, BMI was the only used obesity screening anthropometric parameter, few studies suggested that WHR and LAP were more reliable anthropometric parameters than BMI, particularly in cognitive function screening [52, 58].

The studies included in the present review used MMSE and ACE III as the cognitive assessment tool. However, there are several cognitive screening tools available to screen cognition that cover various neuropsychological domains of cognition such as attention, memory, language, executive function, and orientation [40]. In studies that screened for cognition, MMSE [54, 59], Montreal Cognitive Assessment (MoCA) [19, 52], and Neuropsychological Batteries [8, 28, 58] have been used. The use of a combination of cognitive assessment tools help to discover different neuropsychological domains [40], and it provides a broader understanding of the occurrence of MCI in people with obesity. Furthermore, it will help to identify the best tool for screening MCI in people with obesity.

## Conclusions

Although there was no adequate evidence to estimate the overall effect between MCI and obesity, the available findings in this systematic review support that the prevalence of MCI among people with obesity from the general population is higher. Furthermore, there is a higher potential to observe MCI in obese people compared to normal-weight people. Therefore, early identification of MCI in people with obesity is essential to diminish further deterioration of cognitive function and expand studies for MCI of people with obesity in the general population.

Furthermore, the following suggestions would be important for future studies: using a cognitive screening tool that can screen MCI, incorporate numerous anthropometric parameters to screen obesity rather than limiting to a single anthropometric parameter and, perform subgroup analysis by possible age groups such as young, middle adulthood, older adulthood.

## Data Availability

Data sharing is not applicable to this article as no datasets were generated or analysed during the current study.

## Abbreviations

ACE III: Addenbrooke’s Cognitive Examination
BMI: Body Mass Index
JBI: Jonna Briggs Institute
LAP: Lipid Accumulation Products
MMSE: Mini-Mental State Examination
MCI: Mild Cognitive Impairment
MoCA: Montreal Cognitive Assessment
PRISMA: The Preferred Reporting Items for Systematic Reviews and Meta-Analyses
WHR: Waist-to-Hip Ratio
